# Synergistic Enhancement of Chemotherapy-Induced Cell Death and Antitumor Efficacy against Tumoral T-Cell Lymphoblasts by IMMUNEPOTENT CRP

**DOI:** 10.3390/ijms25147938

**Published:** 2024-07-20

**Authors:** Ana Luisa Rivera-Lazarín, Kenny Misael Calvillo-Rodríguez, Mizael Izaguirre-Rodríguez, José Manuel Vázquez-Guillén, Ana Carolina Martínez-Torres, Cristina Rodríguez-Padilla

**Affiliations:** 1 Laboratorio de Inmunología y Virología, Facultad de Ciencias Biológicas, Universidad Autónoma de Nuevo León, San Nicolás de los Garza 66455, Mexico; 2 LONGEVEDEN S.A. De C.V., Guadalupe 67199, Mexico

**Keywords:** ICRP, chemotherapy, synergism, apoptosis, bone marrow

## Abstract

T-cell malignancies, including T-cell acute lymphoblastic leukemia (T-ALL) and T-cell lymphoblastic lymphoma (T-LBL), present significant challenges to treatment due to their aggressive nature and chemoresistance. Chemotherapies remain a mainstay for their management, but the aggressiveness of these cancers and their associated toxicities pose limitations. Immunepotent CRP (ICRP), a bovine dialyzable leukocyte extract, has shown promise in inducing cytotoxicity against various cancer types, including hematological cancers. In this study, we investigated the combined effect of ICRP with a panel of chemotherapies on cell line models of T-ALL and T-LBL (CEM and L5178Y-R cells, respectively) and its impact on immune system cells (peripheral blood mononuclear cells, splenic and bone marrow cells). Our findings demonstrate that combining ICRP with chemotherapies enhances cytotoxicity against tumoral T-cell lymphoblasts. ICRP + Cyclophosphamide (CTX) cytotoxicity is induced through a caspase-, reactive oxygen species (ROS)-, and calcium-dependent mechanism involving the loss of mitochondrial membrane potential, an increase in ROS production, and caspase activation. Low doses of ICRP in combination with CTX spare non-tumoral immune cells, overcome the bone marrow-induced resistance to CTX cell death, and improves the CTX antitumor effect in vivo in syngeneic Balb/c mice challenged with L5178Y-R. This led to a reduction in tumor volume and a decrease in Ki-67 proliferation marker expression and the granulocyte/lymphocyte ratio. These results set the basis for further research into the clinical application of ICRP in combination with chemotherapeutic regimens for improving outcomes in T-cell malignancies.

## 1. Introduction

T-cell malignancies comprise a group of neoplasms that arise from the expansion of dysfunctional T-cells at different stages of development. T-cell acute lymphoblastic leukemia (T-ALL) is the most common T-cell cancer in children. In contrast, T-cell lymphoblastic lymphoma (T-LBL) accounts for 20% of the non-Hodgkin lymphoma cases in children. Studies have lent strength to the theory that T-LBL and T-ALL may evolve from a common malignant precursor cell [1,2]; moreover, both diseases are aggressive forms of hematological cancers since T-cell’s overall prognosis is poorer than B-cell malignancies [3,4]. Chemotherapies, such as cyclophosphamide (CTX), etoposide (ETO), and anthracyclines such as doxorubicin (DOX) and epirubicin (EPI) remain a potential strategy for T-ALL/T-LBL [5,6,7,8,9]. Several chemotherapies act primarily through the induction of apoptosis beyond distinct targets for these agents in susceptible cancer cells [10]. Also, in high doses, they cause severe secondary effects, such as bone marrow suppression, spleen toxicity [5,6,7,8,9], cognitive impairment, and microglial death [7].

Managing treatment during disease recurrence remains challenging due to chemoresistance, which arises from various mechanisms, including the inherent sensitivity of cancer cells to evade cell death [5,11,12]. Therefore, efforts to overcome resistance have pointed out the use of multi-targeted agents through the assessment of drug combinations, guided by an understanding of the molecular mechanisms underlying cell death. In this regard, recent studies highlighted the advances and the growing relevance of simultaneously blocking multiple pathogenic pathways in B-cell malignancies and lymphoma [13,14]. The multiple targets can belong to the same or different pathways of cell death that converge at a pathway site, resulting in an enhanced effect. Combination therapy works in a synergistic, additive, or antagonistic manner depending on the amount of the drug combination effect, which can be quantified by several models [15]. A substantial amount of evidence uses the combination index (CI) analysis proposed by Chou-Talay, which mitigates uncertainties in identifying effective combination treatments by enabling the scoring of synergistic drug effects [16,17]. Multiple reports provide evidence of combining chemotherapies and immunotherapies [18], which enables a reduction in the toxic effects on healthy cells and enhances efficacy against cancer cells at lower dosages, potentially overcoming chemo-resistance [19].

Immunepotent CRP (ICRP), a bovine dialyzable leukocyte extract, is an immunotherapy reported to exhibit immunomodulatory properties and cytotoxicity against several cancer cell lines [20,21]. The combinational therapy of ICRP with DOX and CTX modified the tumor microenvironment in a murine breast cancer model [22]. Furthermore, the combination of ICRP and oxaliplatin (OXP), induced immunogenic cell death (ICD) in murine melanoma [23]. ICRP was also reported to improve the clinical parameters of breast cancer patients receiving standard chemotherapy [24]. Therefore, ICRP shows potential when combined with various chemotherapies, including CTX, a major chemotherapy used for hematologic malignancies. Thus, in the present study, we investigated the combinatorial effects of a panel of chemotherapies and ICRP treatment on two T-cell malignancies, T-ALL and T-LBL c, chosen for their aggressive nature, poor prognosis, response to therapy, and chemoresistance, focusing on the mechanism of the CTX-ICRP combination and its in vivo effects.

## 2. Results

### 2.1. ICRP, CTX, DOX, EPI, and ETO Induce Tumoral T-Cell Lymphoblasts Cell Death

**Figure 1:**
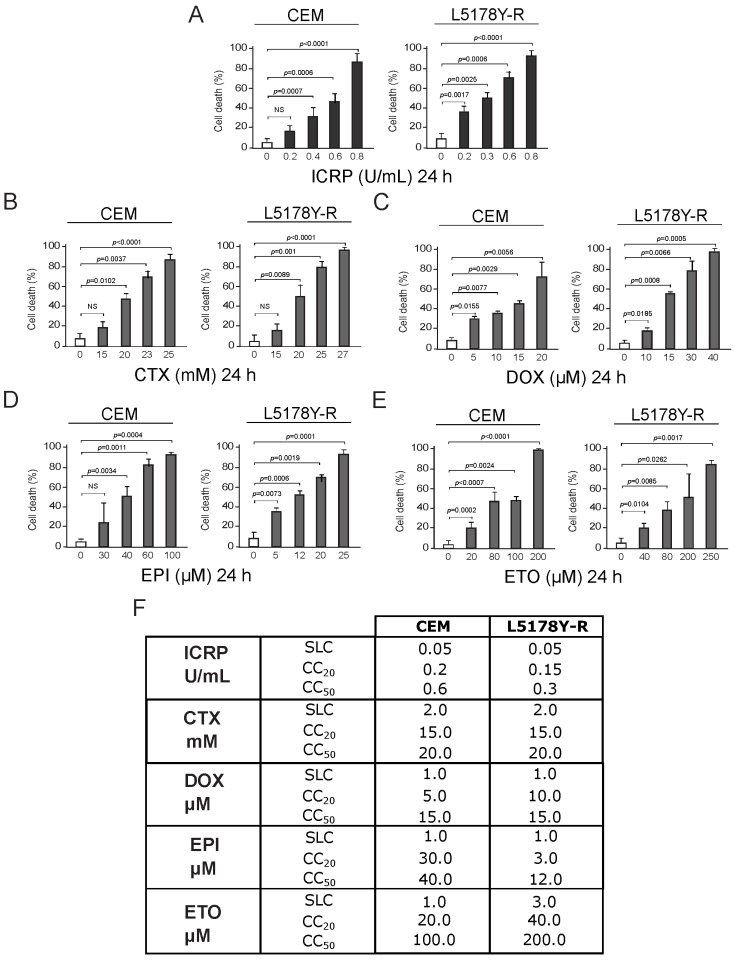
ICRP, CTX, DOX, EPI, and ETO induce cell death in tumoral T-cell lymphoblasts. CEM and L5178Y-R cell lines were treated for 24 h, and biochemical features of cell death were assessed and expressed in percentage (%). Cell death was analyzed by Annexin V/PI staining for (**A**) ICRP-, (**B**) CTX-, and (**E**) ETO-treated cells or only AnnV for (**C**) DOX and (**D**) EPI treatments. (**F**) Sublethal concentration (SLC), cytotoxic concentration that induced cell death of 20% of the cells (CC_20_) and cytotoxic concentration that induced cell death of 50% of the cells (CC_50_) found for IMMUNEPOTENT CRP (ICRP), Cyclophosphamide (CTX), Doxorubicin (DOX), Epirubicin (EPI) and Etoposide (ETO) are summarized for CEM and L5178Y-R cell lines. Graphs are the means ± SD of triplicates from at least three independent experiments. NS was assigned to *p* > 0.05.

CEM and L5178Y-R death was analyzed after ICRP (dark gray) or chemotherapy (light gray) treatment. Results showed that all treatments augment tumoral T-cell lymphoblast cell death as treatment concentration increases (Figure 1A–E). Data show cell death of 20% of the cells (CC_20_) at 0.2 and 0.15 U/mL of ICRP for CEM and L5178Y-R cells, respectively, meanwhile 50% of the cells were dead (CC_50_) at 0.6 and 0.3 U/mL of ICRP (Figure 1A), respectively. On the other hand, CTX CC_20_ was 15 mM for both cell lines while 20 mM CTX was required to induce cell death in 50% of the cell population for both cell lines (Figure 1B). Likewise, DOX CC_20_ was shown at 5 μM for CEM and 10 μM for L5178Y-R, whereas DOX CC_50_ was shown at 15 μM for both cell lines (Figure 1C). Furthermore, EPI CC_20_ was 30 μM for CEM and 3 μM for L5178Y-R, whereas EPI CC_50_ was obtained at 40 μM for CEM and 12 μM for L5178Y-R (Figure 1D). Additionally, 20 μM and 40 μM ETO were the CC_20_, while 100 μM and 200 μM ETO were the CC_50_ of CEM and L5178Y-R, respectively (Figure 1E). CC_20_ and CC_50_ cytotoxic concentrations were found and the sublethal concentration was taken as the highest concentration of each treatment that does not induce notable cell death, for each cell line and treatment. These concentrations are summarized in the table shown in Figure 1F.

Although chemotherapies have different mechanisms of action, we proposed that a potentiated cytotoxic effect could be achieved by combining them with ICRP.

### 2.2. The Combination of ICRP and Chemotherapies Potentiates Cell Death against Tumoral T-Cell Lymphoblasts

Different combination ratios were designed for investigating the effect of several concentrations of ICRP on chemotherapies’ cytotoxicity. The chemotherapies for combination studies were chosen from a panel of chemotherapies (with different mechanisms of action such as alkylating agents and topoisomerase inhibitors) that were able to directly induce cell death as monotherapies in the cell lines tested. In contrast, we discarded the antimetabolites Ara-C and Methotrexate as they were unable to induce 50% cell death in L5178Y cells (Figure S1). First, we used a non-cytotoxic concentration (SLC, sublethal) of ICRP, in combination with the CTX, DOX, EPI, and ETO − CC_50_ of each tumoral T-cell lymphoblasts cell line. To investigate whether chemotherapies affect ICRP cell death, we tested the combination of CC_50_ ICRP with SLC CTX, DOX, EPI, and ETO. To examine the combined effect of equipotent concentrations of both treatments, we tested the combination of CC_20_ of ICRP and CTX or the combination of CC_50_ of both treatments. Moreover, to investigate whether ICRP at a low dose affects chemotherapies’ cell death, we treated cells with CC_20_ ICRP + CC_50_ CTX, DOX, EPI, and ETO.

**Figure 2:**
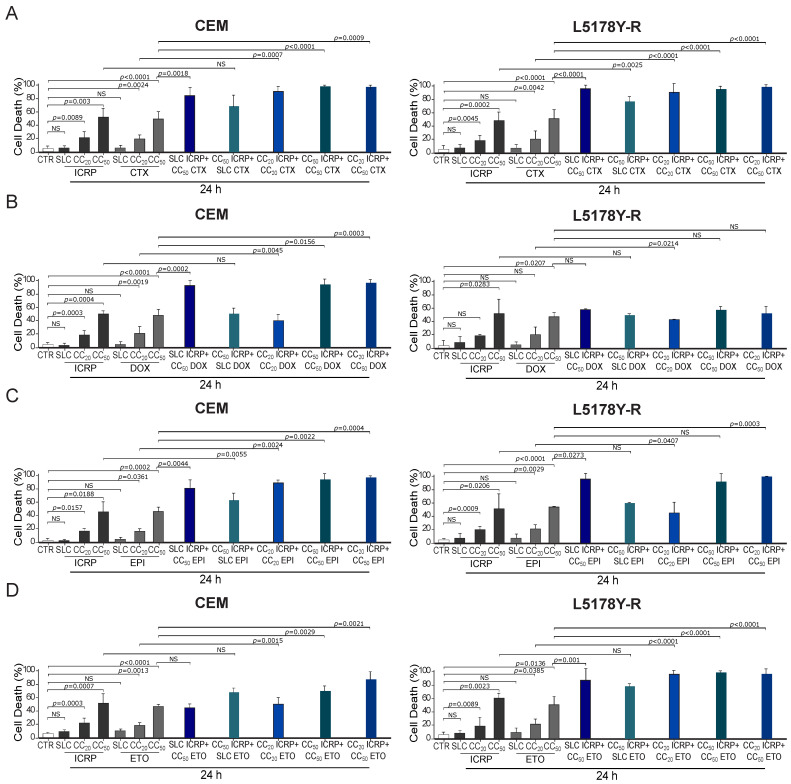
ICRP + chemotherapy-induced cell death in tumoral T-cell lymphoblasts. (**A**–**D**) CEM and L5178Y-R were treated for 24 and analyzed by flow cytometry using Ann/PI staining or Ann alone for DOX and EPI. Cell death induced by (**A**) ICRP, CTX, and its combination, (**B**) ICRP, DOX, and its combination, (**C**) ICRP, EPI, and its combination, and (**D**) ICRP, ETO, and its combination. Graphs are the means ± SD of triplicates from at least three independent experiments. NS was assigned to *p* > 0.05.

As Figure 2A shows, a significant increase in CEM and L5178Y-R cell death compared to single agents was observed, reaching 85% and 96%, respectively, when combining SLC ICRP + CC_50_ CTX. Results showed a non-significant cell death increase in CEM with the combination of CC_50_ ICRP + SLC CTX, whereas this combination induced a significant increase in L5178Y-R death reaching 69% and 77% cell death, respectively. Cell death assessment showed that CC_20_ ICRP + CC_20_ CTX reached 91% cell death in CEM and L5178Y-R. Likewise, CC_50_ ICRP + CC_50_ CTX showed a significant increase in cell death compared to single treatments, reaching 98% and 95% in CEM and L5178Y-R, respectively, and the combination using CC_20_ ICRP + CC_50_ CTX demonstrated 98% cell death in the two cell lines tested. Furthermore, Figure 2B shows that SLC ICRP + CC_50_ DOX demonstrated a significant increase in CEM cell death compared to single treatments, reaching 93%, whereas L5178Y-R showed no significant increase, reaching 59% cell death. The combination using CC_50_ ICRP + SLC DOX induced 50% cell death in CEM and L5178Y-R. A significant increase in CEM and L5178Y-R cell death compared to single agents was observed, reaching 40% and 43%, respectively, when combining CC_20_ ICRP + CC_20_ DOX. When the combination of CC_50_ ICRP + CC_50_ DOX was used we observed a significant cell death increase in CEM, reaching 94%, whereas this combination reached 58% in L5178Y-R cells. The assessment revealed a significant increase in cell death to 97% in CEM when combining CC_20_ ICRP + CC_50_ DOX. Conversely, this combination showed a non-significant increase in cell death in L5178Y-R cells, with 52%.

Moreover, as shown in Figure 2C, a significant increase in cell death occurs when combining SLC ICRP + CC_50_ EPI, demonstrating 81% and 96% cell death in CEM and L5178Y-R, respectively. Results showed a significant cell death augmentation in CEM with the combination of CC_50_ ICRP + SLC EPI reaching 59%, while this combination in L5178Y-R reached 60%. Cell death assessment induced by CC_20_ ICRP + CC_20_ EPI showed a significant increase compared to single treatments, reaching 89% and 46% in CEM and L5178Y-R, respectively. Likewise, CC_50_ ICRP + CC_50_ EPI showed 87% and 91% cell death in CEM and L5178Y-R, respectively, and the combination using CC_20_ ICRP + CC_50_ EPI demonstrated 97% cell death in CEM and 99% in L5178Y-R.

Additionally, Figure 2D shows that SLC ICRP + CC_50_ ETO showed a non-significant increase in CEM cell death compared to ETO alone, reaching 45%, whereas L5178Y-R showed a significant increase, reaching 88% cell death. The combination using CC_50_ ICRP + SLC ETO demonstrated an increase in cell death with 68% and 82% values in CEM and L5178Y-R, respectively. When combining CC_20_ ICRP + CC_20_ ETO, a significant increase in CEM and L5178Y-R cell death was observed compared to single agents, reaching 51% and 95%, respectively. Results showed a significant cell death augmentation in the two cell lines tested when the combination of CC_50_ ICRP + CC_50_ ETO was used, reaching 70% in CEM and 93% in L5178Y-R. Finally, the assessment showed a significant increase in cell death to 87% in CEM and 96% in L5178Y-R when combining CC_20_ ICRP + CC_50_ ETO.

### 2.3. The Combination of ICRP with Chemotherapy Induces a Synergistic Cytotoxic Effect Allowing a Reduction in Chemotherapy Doses

To correctly define whether the combined effect is superior to the single drugs, we used the combination index (CI) to quantify the drug interaction effect induced by ICRP in combination with each chemotherapy by the software Compusyn. Table 1 shows the CI values obtained from all the tested combinations, revealing a synergistic effect (CI < 1) by all the chemotherapies and ratios tested. Nevertheless, the highest synergic effect, according to the CI values shown in both cell lines, was obtained from the combinations of ICRP with CTX.

Furthermore, when looking for a decreasing toxicity in single drugs, as the combined effect is higher than monotherapy, we calculated the degree of chemotherapy dosage reduction by drug reduction index (DRI). All the chemotherapies tested showed DRI values above 1 reaching up to 1724.07, indicating a favorable dose reduction. DRI values are summarized in Table 2.

Considering CTX demonstrated the greatest synergistic effect across both cell lines and a favorable reduction in DRI values, combinations involving SLC ICRP + CC_50_ CTX, CC_50_ ICRP + CC_50_ CTX and CC_20_ ICRP + CC_50_ CTX combinations were chosen to further determine several biochemical features of ICRP + CTX cell death, assessing the main characteristics elicited by each monotherapy.

### 2.4. The Combination of ICRP with CTX Induces Mitochondrial Alterations in Tumoral T-Cell Lymphoblasts

**Figure 3:**
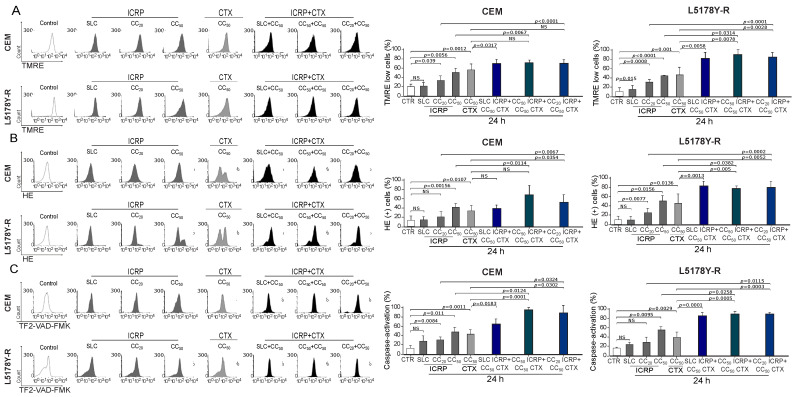
ICRP + CTX cell death-induced mitochondrial alterations in tumoral T-cell lymphoblasts. Cells were treated with ICRP + CTX in distinct ratios for 24 h and analyzed by flow cytometry. Representative histograms and graphs from (**A**) loss of mitochondrial membrane potential, (**B**) ROS production, and (**C**) caspase activation measured using TMRE, HE, and TF2-VAD-FMK staining, respectively, in CEM and L5178Y-R. Graphs are the means ± SD of triplicates from at least three independent experiments. NS was assigned to *p* > 0.05.

The right panel of Figure 3A shows a significant increase in the loss of mitochondrial membrane potential assessment by SLC ICRP + CC_50_ CTX in CEM and L5178Y-R reaching 75% and 82%, respectively, whereas L5178Y-R also showed a significant increase with CC_50_ ICRP + CC_50_ CTX and CC_20_ ICRP + CC_50_ CTX-treatment (86–88%) compared to CTX monotherapy. Likewise, CEM CC_20_ ICRP + CC_50_ CTX-treated cells showed 55% ROS production, and L5178Y-R at all the combination ratios showed a significant increase in ROS production compared to CTX alone, demonstrated by up to 82% HE+ cells (Figure 3B). Additionally, a significant increase in caspase activation was observed after SLC ICRP + CC_50_ CTX, CC_50_ ICRP + CC_50_ CTX, and CC_20_ ICRP + CC_50_ CTX treatment in CEM and L5178Y-R, compared to single agents (Figure 3C).

### 2.5. The Combination of ICRP with CTX Induces Cell Death Involving Caspases, ROS Production, and Calcium Augmentation in Tumoral T-Cell Lymphoblasts

**Figure 4:**
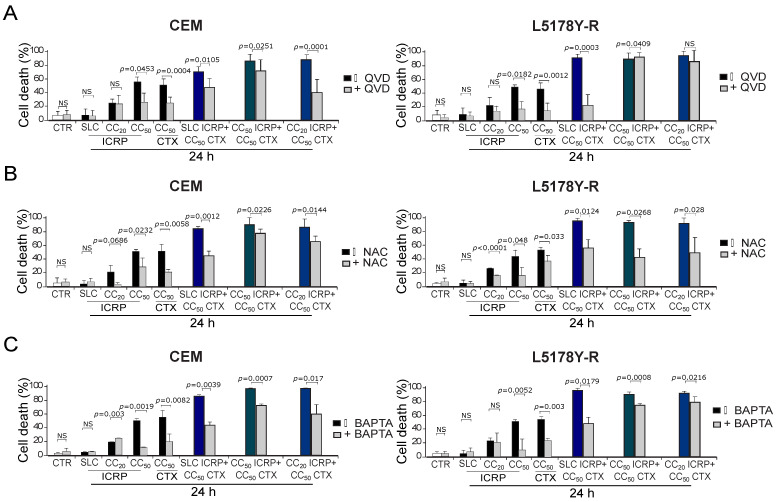
ICRP + CTX cell death effectors in tumoral T-cell lymphoblasts. Cells were treated with (**A**) QVD, (**B**) NAC, or (**C**) BAPTA for 30 min before treatment with ICRP + CTX in distinct ratios for 24 h, and cell death was analyzed by flow cytometry. Graphs from AnnV/PI measurement of CEM (**left** panel) and L5178Y-R (**right** panel). Bars are the means ± SD of triplicates from at least three independent experiments. NS was assigned to *p* > 0.05.

We aimed to investigate the effectors of ICRP + CTX cell death. For this, we analyzed the caspase dependence using the pan-caspase inhibitor QVD. We found that QVD diminished cell death induced by CC_50_ ICRP and CC_50_ CTX in the two cell lines; also in CEM, QVD diminished the cell death in the different combination ratios tested. Whereas, in L5178Y-R, QVD inhibited cell death when cells were treated with SLC ICRP + CC_50_ CTX and CC_50_ ICRP + CC_50_ CTX, but not with the combination CC_20_ ICRP + CC_50_ CTX (Figure 4A). Furthermore, after using the antioxidant NAC, cell death diminished significantly when cells were treated with all the combination ratios tested in both cell lines (Figure 4B). Additionally, pre-treatment with the extracellular calcium chelator BAPTA decreased cell death induced by CC_50_ ICRP and CC_50_ CTX, as well as all the combination ratios tested in both cell lines (Figure 4C).

### 2.6. The Combination of ICRP with CTX Does Not Potentiate CTX Cell Death in Non-Tumoral Immune System Cells and Protects Bone Marrow Cells from CTX Cell Death

**Figure 5:**
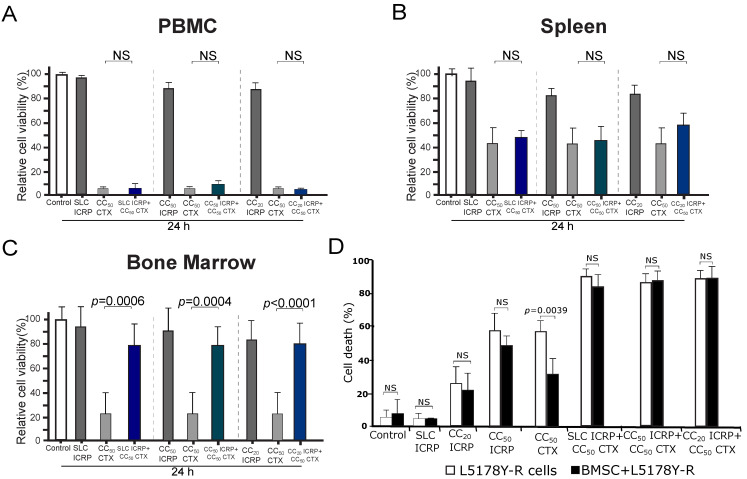
ICRP + CTX cell death in non-tumoral immune system cells and tumoral cells in the presence of BMSC environment. PBMC (**A**), spleen (**B**), and bone marrow cells (**C**) were treated for 24 h with ICRP, CTX, and their combination, analyzed by flow cytometry using Ann/PI staining, and expressed as relative cell viability by the exclusion of Ann V/PI positive cells considering control cells as 100% cell viability. (**D**) Cell death induced by ICRP, CTX, and its combination in L5178Y-R co-cultivated with bone marrow stromal cells (BMSC) and analyzed by flow cytometry. NS was assigned to *p* > 0.05.

To evaluate if the combination of ICRP with CTX could also potentiate the cytotoxicity of non-tumoral immune system cells, we chose the highest cytotoxic concentration used in the tumoral cells to investigate the cytotoxicity of this combination in peripheral blood mononuclear cells (PBMC), splenocytes, and bone marrow cells. As Figure 5 shows, ICRP is not cytotoxic to PBMC (Figure 5A), spleen (Figure 5B), and bone marrow cells (Figure 5C) as only a low relative cell viability reduction was observed at CC_50_ ICRP of CEM (0.6 U/mL, 17% reduction). In contrast, CC_50_ CTX (20 mM) induced a strong reduction in cell viability in all the non-tumoral immune system cells, ranging from 57% to 94% reduction. Interestingly, any of the combination ratios tested increased this reduction in cell viability. Importantly, we observed a significant increase in the relative cell viability of bone marrow cells when treated with all the combination ratios tested, compared to CTX alone. This indicates that ICRP protects against cell death in bone marrow cells.

### 2.7. The Combination of ICRP with CTX Overcomes Cell Death Resistance Induced by Bone Marrow Stromal Cells

Next, we assessed whether ICRP + CTX-induced cell death could be protected by the survival stimuli provided by the bone marrow microenvironment [25]. In Figure 5D, while cell death induced by CC_20_ and CC_50_ ICRP persisted even when L5178Y-R were cocultured with BMSC, the presence of BMSC inhibited cell death in CTX-treated cells. In contrast, the cell death induced by the combination of ICRP + CTX remained unchanged even when using SL concentrations of ICRP (Figure 5D).

### 2.8. The Combination of ICRP with CTX Has an Antitumor Effect against T-Cell Lymphoma

**Figure 6:**
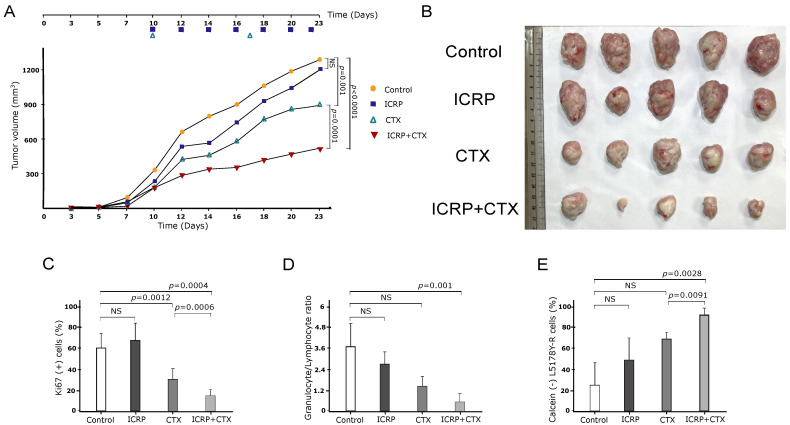
ICRP + CTX induces an antitumor effect against tumoral T-cell lymphoblasts. Female BALB/c mice (n = 5 per group) were inoculated s.c. with 1 × 10^6^ L5178Y-R viable cells. When the tumor reached 100–120 mm^3^ after inoculation, mice were treated with 2 U/mL i.p. ICRP (purple squares) every two days, 125 mg/kg CTX i.p, weekly (green triangles), or the combination of ICRP + CTX (inverted red triangles). Control mice (yellow circles) were treated with 100 μL sterile water for injection. Data are shown in (**A**) graph of tumor volume, (**B**) tumor size photograph, (**C**) Ki67 in tumor cells analyzed by flow cytometry, (**D**) granulocyte/lymphocyte ratio obtained from hematic biometry and (**E**) splenocytes cytotoxicity of mice treated with ICRP, CTX, or its combination against L5178Y-R stained with calcein-AM and analyzed by flow cytometry. NS was assigned to *p* > 0.05.

As Figure 6A shows, female L5178Y-R-bearing mice were treated with a low dose of ICRP, CTX, and their combination. Treatment with two units of ICRP every two days led to a moderate decrease in tumor volume, whereas weekly injections of 125 mg/kg CTX resulted in a significant decrease in tumor volume compared to the control (vehicle-treated group). However, when the low dose of ICRP was combined with CTX, tumor volume significantly diminished compared to CTX monotherapy. This reduction in tumor volume is consistent with the tumor size shown in Figure 6B.

Additionally, as shown in Figure 6C, tumor cells from the control and ICRP groups exhibited a high percentage of the Ki-67 proliferation marker. In contrast, the CTX group showed a decrease in the percentage of Ki-67, which was further reduced in the ICRP + CTX group compared to CTX monotherapy.

A hematic biometry was conducted after treatment, and the granulocyte/lymphocyte ratio was determined. It was observed that this ratio remained unchanged in the peripheral blood of ICRP- and CTX-treated mice compared to control mice. However, the granulocyte/lymphocyte ratio was significantly decreased only in the ICRP + CTX-treated group (Figure 6D). Furthermore, to analyze the specific cytotoxicity of immune cells against cancer cells after treatment, we assessed the cytotoxicity of splenocytes to L5178Y cells. We observed that only splenocytes obtained from ICRP + CTX-treated mice induced a significant increase in L5178Y-R cell cytotoxicity, as evidenced by the loss of calcein staining (Figure 6E).

## 3. Discussion

Chemotherapies are well-known apoptosis inducers and exhibit significant immunosuppressive effects on various organs, including bone marrow, spleen, and the central nervous system [5,6,7,8,9,10]. Immunepotent CRP (ICRP), a bovine dialyzable leukocyte extract, displays selective cytotoxicity against several solid and hematologic cancers by inducing ROS-dependent apoptosis in T-cell acute lymphoblastic leukemia (T-ALL) cells, leading to nuclear and mitochondrial damage [20,26]. This study reported the first use of ICRP in conjunction with chemotherapy to enhance cytotoxicity against T-ALL and T-LBL, which are often resistant to conventional treatments [27,28]. Our findings revealed that combining ICRP with chemotherapy significantly boosts cytotoxicity in T-cell lymphoblasts, showing potential for enhanced antitumor effects. The concept of combination therapy was pioneered by Frei, Holland, and Freireich, who developed the first chemotherapy regimen for ALL [29]. In subsequent studies, there were combined doses of CC_50_ cisplatin after 4-hydroperoxycyclophosphamide treatment, achieving up to 85% inhibition of leukemic cell viability [30], similar to our results of 84–96% cell death using sublethal doses of various chemotherapies combined with ICRP.

Our study indicated that synergistic cytotoxic effects are enhanced by combining ICRP with chemotherapy. There have been reported synergistic cytotoxic effects induced by combinations of low doses of chemotherapies with other treatments, such as combinations of CC_10_ nutlin-3a with CC_20_ doxorubicin (DOX), CC_25_ chlorambucil (CLB), or CC_15_ fludarabine (FLU), which showed 50% to 65% cell death in B-cell chronic lymphocytic leukemia patient’s samples [31]. These results are similar to our findings when using sublethal doses of CTX, DOX, EPI, or ETO, combined with CC_50_ ICRP, where cell death reached 50% to 82%. On the other hand, using a sublethal inhibitory concentration of nelarabine (nela) in combination with the inhibitory concentration 15 (IC_15_) of ZSTK-474, induced a 25% cell viability inhibition of T-ALL patient’s samples [32]. These results are different from the ones observed when we combined suboptimal (CC_20_) concentrations of ICRP and CTX, DOX, EPI, or ETO as these combinations reached up to 95% cell death, demonstrating a synergistic effect of CI values lower than 1.0. Furthermore, improved efficacy in terms of cytotoxicity was obtained by treatment using CC_20_ nela plus CC_50_ ZSTK-474, inducing 60% cell viability inhibition. Remarkably, CC_20_ ICRP plus CC_50_ CTX, DOX, EPI, or ETO improved the cell death induced by monotherapies, showing 87% to 98%. These data underline the potential of ICRP in potentiating chemotherapy-induced cell death, even when used at non-lethal or suboptimal concentrations.

Combinations of several agents such as BV6, a bivalent SMAC mimetic, and nela, with chemotherapies at ratios using equipotent concentrations of both treatments, revealed higher cytotoxicity induced by ICRP plus CTX, DOX, EPI, or ETO. For instance, IC_50_ BV6 combined with IC_50_ CTX showed a decrease in cell viability to 20% in primary ALL cells [12]. On the other hand, a combination that included CC_40_ nela and CC_40_ ZSTK-474 against ALL cells reached 70% inhibition of cell viability. When we combined CC_50_ ICRP+ CC_50_ of each chemotherapy, our results produced up to 98% cell death, leading to CI values representing a synergistic cytotoxic effect [32].

Combination therapy with synergistic or additive effects may produce a more potent cytotoxic effect in lower doses of each monotherapy. We observed CI values reaching 0.00745–0.99682, showing a stronger synergism as well as more favorable DRI values (1.59690–1724.07) than shown previously by Hosseini M. and colleagues which combined different ratios of carfilzomib (cfz) and dexamethasone (Dex) against MOLT-4, a T-ALL cell line, and obtained 0.983–0.749 in CI values and 2.243–41.951 [33]. Furthermore, our results regarding the CI and DRI values are also different from the ones reported by Hassani S et al., who combined azidothymidine (AZT) and arsenic trioxide (ATO) in different ratios and found a reduction in the ATO cytotoxicity, showing an antagonistic effect with CI values of 1.21–5.54 and non-favorable or non-dose-reduction for ATO with 0.46–1.32 DRI values [34]. These data emphasize the potential of ICRP in boosting the effectiveness of existing chemotherapy protocols in T-ALL and T-LBL, particularly at suboptimal concentrations that are less toxic to healthy cells.

A synergistic effect could be triggered by actions on multiple targets that reside in the same or different pathways, negative regulation of counteractive actions, facilitating actions, or due to complementary actions [15]. Our data showed that both ICRP and CTX induce the loss of mitochondrial membrane potential, an increase in ROS production, and caspase activation. These effects were significantly augmented when the treatments were combined, compared to each treatment alone, in most of the combination ratios tested in both cell lines. Therefore, it seems that the increased cytotoxic effects of ICRP + CTX could be at least in some part due to the enhancement of mitochondrial alterations which can initiate cell death, similar to the results previously reported by combining Cfz + Dex which showed a significant increase in caspase 3, BAX and BCL2 gene expression in a T-ALL cell line compared to monotherapy [33]. Moreover, we further identified the role of caspase activation, ROS production, and intracellular calcium overload during cell death. As previously reported, ICRP and CTX cell death rely on caspase activation and ROS production, whereas ICRP cell death also depends on the increase in intracellular calcium levels in T-ALL [5,6,20,35]. Yet, here we first reported the relevance of an increase in the intracellular calcium for CTX-mediated cell death as it was previously described for cardiomyocyte toxicity [36]. ICRP + CTX showed mostly caspase-dependent, ROS-dependent, and Calcium-dependent cell death. However, we could note that even if ICRP alone induces caspase-dependent cell death, caspases were dispensable when using CC_20_ ICRP alone, such independence was maintained in the combination CTX + ICRP CC_20_ in L5178Y-R cells. We previously demonstrated that in breast cancer cell lines (MCF-7, MDA-MB231, and 4T1 cells) ICRP induces caspase-independent cell death, and the combination of ICRP + CTX maintains such caspase-independent cell death; however, ROS dependence was not assessed [37]. Other ROS- and caspases-dependent cell death modalities have been shown by the combination of bortezomib with PCI-24781 (an HDAC inhibitor) synergized against a Hodgkin and a non-Hodkin lymphoma cell line [38]. The combination of phytosphingosine and ionizing radiation in a T-cell lymphoma cell line resistant to radiation also involved the loss of mitochondrial membrane potential and resulted in a caspase-independent mechanism [39].

Conventional chemotherapies can be toxic to healthy cells, leading to multiple side effects, including a reduction in the immune system by affecting lymphoid organs such as bone marrow and the spleen [5,6,7,8,9]. Although combination therapy can be toxic, the low therapeutic dosage required of each drug may prevent the toxic effects on healthy cells, while potentiating the cytotoxic effects on cancer cells. This may occur if one drug in the combination regimen is non-cytotoxic to healthy cells [19], as is the case in several immunotherapies, which show immunomodulatory activities but also present cytotoxic activities against cancer cells [40]. Although CTX induced variable cytotoxic effects in non-tumoral immune system cells, ICRP was not toxic. When combining both treatments using the concentrations and combination ratios tested in tumoral cells, ICRP + CTX did not demonstrate an increase in the cytotoxic effect of CTX in PBMC and spleen cells, but also, ICRP inhibited the CTX toxicity induced in bone marrow cells. This cytoprotection observed in bone marrow cells is in accordance with previous reports of our research group, where it was demonstrated that ICRP was able to induce in vivo bone marrow cell protection after 5-Fluorouracil treatment by reducing ROS production [41]. Other naturally derived products, such as a mixture of honeybee compounds, showed the in vivo amelioration of the cytotoxic effects of CTX in bone marrow cells, sperm, and the liver when used in combination with CTX [42]. However, the Janus-like effect of ICRP, where on one hand it is cytotoxic to cancerous cells and cytoprotective to bone marrow cells, could be related to its capacity to induce ER stress. This was demonstrated in T-ALL, where it induces ER stress through ER-Ca^2+^ mobilization and prosurvival autophagosome formation [35]. It has been demonstrated that depending on the duration and intensity of the stress, ER stress can switch from protection to cell death induction [43], and even in the presence of autophagy, the same molecular cascades that initially support the cytoprotection shift to a cytotoxic mode and ultimately promote cell death [44]. Here, we observed that Ca^2+^ mobilization in CTX + ICRP treatment is important for cell death induction, and it has been demonstrated that T-ALL cells upregulate the machinery and signaling molecules associated with ER stress and autophagy [43,45,46]. On the other hand, autophagosomes usually serve as a cell antioxidant pathway [47], which can be linked to the antioxidant activity previously observed in bone marrow cells of mice treated with ICRP. Thus, it is plausible that the mechanism induced by ICRP is in the tightrope between cytoprotective effects in bone marrow cells and the cytotoxic effect observed in cancer cells. This overexpressed ER stress machinery in leukemic cells, which usually promotes prosurvival mechanisms when activated by ICRP treatment, could trigger perturbations that exceed cellular repair capacities leading to cell death. However, further studies on the precise role of ICRP in cytoprotection and the comparison between non-tumor and tumor cells must be performed to better understand this Janus-like role.

Bone marrow niches support stem cells and their progeny, protecting malignant cells from chemotherapy and ultimately contributing to the recurrence of hematological malignancies [25]. Our results revealed that CTX cell death is modulated by BMSC; in contrast, ICRP-induced cell death remained unchanged under these conditions. Also, ICRP + CTX overcame this CTX resistance, even when the combination included SL concentrations of ICRP. Similar results were reported by the peptide RCP168 which partially inhibited stroma-mediated resistance of Jurkat cells (T-ALL) to cytarabine (Ara-C) cell death [48]. Further analysis should be performed to identify the molecular mechanism by which ICRP + CTX overcomes the BMSC-mediated CTX resistance.

The combination strategies are based on sequential or concurrent therapy [49]. Our results show that concurrent therapy, initiating the administration of ICRP when beginning chemotherapy treatment, improved the tumor volume and the proliferation marker reduction induced by CTX alone in T-cell lymphoblastic lymphoma-bearing mice. A previous clinical trial in non-small cell lung cancer ICRP was administered on the third day after chemotherapy and cisplatin treatment. In this study, no changes in tumor size were observed when ICRP was administered, with respect to conventional treatment alone, although ICRP showed a beneficial effect in lymphocyte numbers and improved the Karnofsky score in patients [50]. Later, a clinical trial in breast cancer patients was performed using ICRP starting with 1-week administration prior to chemotherapy, with continued administration during the chemotherapy cycle and up to 1 month after the completion of chemotherapy. ICRP also showed a beneficial effect in lymphocyte numbers and improved Karnofsky score, but this schema also achieved better complete response percentages in stage III and IV patients, and the regression of metastatic lesions was obtained in less time than in the control group [24]. These results point out that administering ICRP at the same time as or before chemotherapy could be the best option in a conventional treatment for T-ALL or T-LBL. However, clinical trials must be performed to confirm this.

In previous research, when CTX was combined with Interferon type I (IFN-I) in vivo, it delayed tumor development and prevented 60% of mice bearing two types of T-cell lymphoma, whereas CTX or IFN alone did not prevent tumor-bearing mice [51]. Furthermore, mice surviving after IFN + CTX could generate immunologic memory, as hypothesized by our results as splenocytes from mice treated with ICRP + CTX showed cytotoxic capacity against the T-LBL cell line. Additionally, the significant decrease in the granulocyte/lymphocyte ratio shown by ICRP + CTX indicates a better anti-tumor efficiency as an elevated ratio seems to be associated with tumor progression and metastasis, perhaps because granulocytes compromise the natural antitumor function of lymphocytes [52].

**Figure 7:**
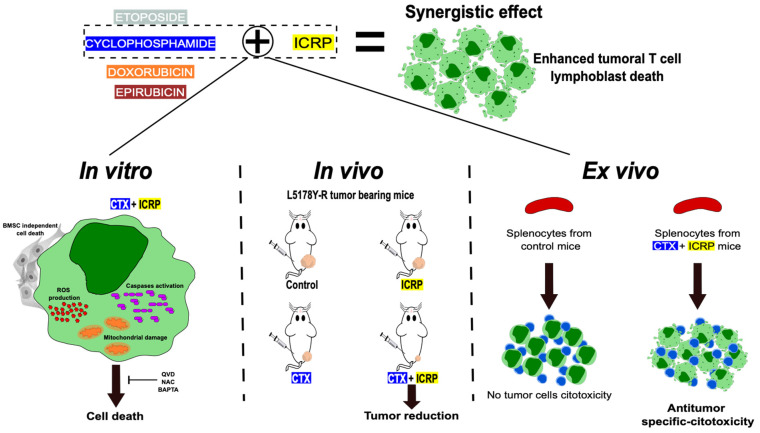
Immunepotent CRP synergistic enhances chemotherapy-induced cell death against tumoral T-cell lymphoblasts. When Immunepotent CRP (ICRP) is combined with Cyclophosphamide (CTX) it enhances ROS production, caspase activation, mitochondrial damage and induces cell death even in the presence of protecting bone marrow stromal cells. The cell death induced depends on caspases, ROS, and calcium. In vivo, the combination of ICRP and Cyclophosphamide enhance the reduction in tumor volume, leading ex vivo to the specific antitumor cytotoxicity induced by splenocytes of the treated mice.

Overall, throughout this study, we demonstrated that combining ICRP with chemotherapy synergically enhances cytotoxicity against T-cell lymphoblasts even when ICRP was used at non-lethal or suboptimal concentrations, whereas ICRP + CTX overcomes the bone marrow-induced resistance to CTX cell death. Furthermore, ICRP improves the CTX antitumor effect in vivo and promotes cancer cell killing by splenocytes ex vivo (Figure 7). These results set the basis for further research into the clinical application of ICRP in combination with chemotherapeutic regimens for improving outcomes in T-cell malignancies.

## 4. Materials and Methods

### 4.1. Cytotoxic Agents, Cell Culture Mediums, and Inhibitors

Cells were cultured in RPMI-1640 supplemented with heat-inactivated-10% fetal bovine serum (FBS) and 1% penicillin-streptomycin (GIBCO by Life Technologies, Grand Island, NY, USA) referred to now as complete RPMI. The Laboratory of Immunology and Virology from the School of Biological Sciences produced IMMUNEPOTENT CRP (ICRP). One unit of ICRP contains 24 mg of peptides obtained from 15 × 10^8^ leukocytes. The general characterization of ICRP was previously reported [53,54,55], where physical, bromatological, chemical, and in silico analyses were reported. ICRP and Cyclophosphamide (Cryofaxol from Cryopharma; Tlajomulco de Zuñiga, Jalisco, Mexico) were dissolved in complete RPMI. Doxorubicin (DOX), Epirubicin (EPI) (Farmorubicin RD^®^, purchased from Pfizer, Mexico City, Mexico), and Etoposide (ETO, Cavep^®.^ from Accord Farma, Mexico City, Mexico) were dissolved in sterile water for injection as appropriate. The antioxidant, N-acetyl-L-cysteine (NAC), was dissolved in water to a final concentration of 500 mM. The pan-caspase inhibitor, QVD.opH (QVD, 1 mM), and the extracellular calcium chelator, BAPTA (50 μM), were dissolved in dimethyl sulfoxide (DMSO) and were incubated for 30 min before treatment. All the solutions were wrapped in foil and stored according to the manufacturer’s instructions.

### 4.2. Cell Culture

The CEM cell line, female human T-cell acute lymphoblastic leukemia (ATCC CCL-119), and L5178Y-R, murine T-cell lymphoblasts (ATCC CRL-1722), were obtained from the American Type Culture Collection (ATCC) and maintained according to its standards in a humidified incubator at 37 °C and 5% CO_2_. Cells were maintained in 25 cm^3^ cell culture flasks (CORNING Enterprises, Corning, NY, USA) containing complete RPMI.

### 4.3. Ethical Consideration

All experiments were reviewed and approved by the Ethical Committee (CEIBA) of the College of Biological Sciences at the UANL: CEIBA-2020-015. For animal samples, all experiments were performed following the Mexican regulation NOM-062-ZOO-1999 and were designed according to the Arrive guidelines for animal care and protection [56]. The procedures in our study involving human samples were conducted in accordance with the Helsinki Declaration.

### 4.4. Animals

The animal house at the Universidad Autónoma de Nuevo León, Mexico, supplied female BALB/c mice (eight-to-ten-week-old; 25 ± 5 g weight). Mice were housed in plastic cages in groups of five, and seven days were given to acclimate to the housing facility. Animals were maintained at 21 ± 3 °C, 55% ±10% humidity, and 12 h light/dark cycle. Mice were provided with rodent maintenance food (LabDiet, St. Louis, MO, USA) and water ad libitum, and health status was monitored daily. Mice were randomly assigned to different groups for all the studies.

### 4.5. Lymphoid Cell Isolation

Male mice (n = 4) were anesthetized using 100 mg/kg sodium pentobarbital (CHEMINOVA, Mexico City, Mexico) and sacrificed by cervical dislocation. Then, the spleen, femur, and tibia were obtained. The spleen was filtered through a cell strainer (70 μM) with PBS. Bone marrow cells were obtained by flushing the femur and tibia into complete RPMI. All cells were maintained at 2 × 10^5^ per well in complete RPMI at 37 °C in a 5% CO_2_ atmosphere.

### 4.6. Peripheral Blood Mononuclear Cells (PBMC) Isolation

After obtaining written informed consent, human PBMC isolation from healthy donors was performed by gradient centrifugation using Ficoll-Paque™ PLUS (GE Healthcare, Chicago, IL, USA). Cell layers were obtained from which the population corresponding to PBMC was taken. Cells were maintained in complete RPMI at 2 × 10^5^ cells per well at 37 °C in a 5% CO_2_ atmosphere.

### 4.7. Cell Death Analysis

Cells (5 × 10^5^ cells/mL) were exposed to ICRP (0.2–0.8 U/mL), CTX (15–27 mM), DOX (5–40 μM), EPI (5–100 μM), ETO (20–250 μM), and the cytotoxic concentrations (CC) used for the combination treatment were obtained. For the following assays, different combination ratios of ICRP + CTX, ICRP + DOX, ICRP + EPI, and ICRP + ETO were used to treat cells for 24 h in 96-well dishes (Life Sciences, Darmstadt, Germany). After incubation, cells were collected and washed with PBS and suspended in 100 μL of binding buffer (10 mM HEPES/NaOH pH 7.4, 140 mM NaCl, 2.5 mM CaCl_2_) containing Annexin-V-APC (AnnV, 1 μg/mL, BD Pharmingen, San Jose, CA, USA) and propidium iodide staining (PI, 0.5 μg/mL, MilliporeSigma, Eugene, OR, USA) to measure cell death with BD Accuri c6 flow cytometer (Becton Dickinson, Franklin Lakes, NJ, USA) and analyzed using FlowJo 10.7.2 Software (BD Biosciences, Ashland, OR, USA).

### 4.8. Pharmacological Inhibition of Cell Death Analysis

Before treatment with ICRP + CTX, cells were treated for 30 min with or without 1.5 µM QVD, 0.25 mM NAC, or 50 μM BAPTA for cell death inhibition. After 24 h, cells were obtained and washed with PBS twice, and suspended in 100 μL of binding buffer (10 mM HEPES/NaOH pH 7.4, 140 mM NaCl, 2.5 mM CaCl_2_) containing Annexin-V-APC (1 μg/mL, BD Pharmingen, San Jose, CA, USA) and 0.5 μg/mL propidium iodide (PI, MilliporeSigma, Eugene, OR, USA) to determine cell death using a BD Accury c6 flow cytometer (Becton Dickinson, Franklin Lakes, NJ, USA). FlowJo Software was used to analyze data (BD Biosciences).

### 4.9. Stromal Bone Marrow Cells’ Protection Analysis

Bone marrow cells were obtained as mentioned above and plated in a flat plate for 48 h. Adherent cells were taken as stromal cells. L5178Y-R was then incubated with the bone marrow stromal cells (BMSC) and its supernatant at a 1:10 ratio (tumor to BMSC) prior to ICRP, CTX, and ICRP + CTX treatment as mentioned above. Cell death was then measured as described previously.

### 4.10. ROS Production Analysis

Quantification of ROS production was performed using 2.5 μM Hydroetidine (HE) staining (Invitrogen, St. Louis, MO, USA). Cells (5 × 10^5^ cells/mL) were exposed to ICRP, CTX, and their combination in 96-well dishes (CORNING) for 24 h. Cells were then harvested and washed with PBS before staining incubation. HE was incubated for 30 min at 37 °C and then washed with PBS for assessment by flow cytometry and analyzed as described above.

### 4.11. Mitochondrial Membrane Potential Analysis

In 5 × 10^5^ cells/mL plated in 96-well dishes (CORNING), treated as mentioned before, and then collected, we performed tetramethyl rhodamine ethyl ester staining analysis (TMRE, 125 nM, Sigma-Aldrich, St. Louis, MO, USA) which was incubated at 37 °C for 30 min to determine loss of mitochondrial membrane potential. Then, cells were washed with PBS to measure the loss of TMRE-fluorescence by flow cytometry as described above.

### 4.12. Caspase Activity Assay

TF2-VAD-FMK, the Generic Caspase Activity FMK staining kit staining (Abcam, Cambridge, UK) was used to assess caspase activity in cells (5 × 10^5^ cells/mL) that were treated with ICRP, CTX, and ICRP + CTX-combinations for 24 h, according to manufacturer’s instructions. Analyses were performed by flow cytometry as described above.

### 4.13. Tumor Establishment and Treatment

L5178Y-R cells (1 × 10^6^) were suspended in 100 μL PBS and injected into the female mice left hind s.c. Three times per week, the tumor volume and mice weight were measured using a caliper (Digimatic Caliper Mitutoyo Corporation, Kanagawa, Japan) and a digital scale (American Weigh Scale-600-BLK, Atlanta, GA, USA). When the tumor reached 100–120 mm^3^ after inoculation, mice (n = 5 per group, assigned randomly) were injected with 2 U i.p. every two days, 125 mg/kg CTX i.p., weekly, or the combination of ICRP + CTX. Control mice were treated with 100 μL sterile water for injection. All treatments were dissolved in sterile water for injection. The following formula was used to determine tumor volume: tumor volume (mm^3^) = (Length × width^2^)/2. Twenty-three days after inoculation of tumor cells, mice were anesthetized as mentioned above, blood was obtained by cardiac puncture for hematic biometry, from which the granulocyte/lymphocyte ratio was determined, and mice were then euthanized by cervical dislocation. Tumor and spleen were obtained and weighed.

### 4.14. Splenocytes + L5178Y-R Co-Culture

L5178Y-R was stained with 0.1 mg/mL Calcein-AM (BD biosciences, San José, CA, USA) for 30 min at 37 °C and 5% CO_2_. Cells were then washed twice with PBS. Thus, splenocytes (obtained as previously described) were added in a 44:1 (splenocytes to tumor) ratio. Co-culture was maintained at 37 °C and 5% CO_2_ for 24 h and calcein-negative L5178Y-R cells were measured by flow cytometry.

### 4.15. Ki67 Analysis

Dissected tumors were macerated and filtered through a cell strainer (70 μM) with PBS and tumor cells (1 × 10^6^) were fixed then in ethanol dropwise gradient (50% to 70%) while vortexing and incubated at −20 °C overnight. Cells were washed twice and analyzed using Ki-67 (Alexa Fluor 647 anti-human Ki-67 Antibody, BioLegend, San Diego, CA, USA).

### 4.16. Statistical Analysis

Triplicate determinations from at least three independent experiments were presented as means ± SD in graphs. Results were analyzed by GraphPad Prism software (San Diego, CA, USA), using paired Student’s *t*-tests for in vitro studies, and two-tailed unpaired Student’s-*t*-tests and Mann–Whitney tests for the ex vivo and in vivo studies, considering statistical significance as *p* < 0.05.

## Figures and Tables

**Table 1:** CI values compilation from the combinations of ICRP with chemotherapies in tumoral T-cell lymphoblasts.

Cytotoxic Concentration		Combination Index (CI)			
**ICRP**	**CTX**	**CEM**	**Interpretation**	**L5178Y-R**	**Interpretation**
SLC	CC_50_	0.13084	Synergism	0.03732	Synergism
CC_20_	CC_20_	0.11256	Synergism	0.11560	Synergism
CC_20_	CC_50_	0.03383	Synergism	0.02743	Synergism
CC_50_	SLC	0.38717	Synergism	0.28102	Synergism
CC_50_	CC_50_	0.02802	Synergism	0.09422	Synergism
**ICRP**	**DOX**	**CEM**	**Interpretation**	**L5178Y-R**	**Interpretation**
SLC	CC_50_	0.08249	Synergism	0.45562	Synergism
CC_20_	CC_20_	0.99682	Synergism	0.96383	Synergism
CC_20_	CC_50_	0.05606	Synergism	0.94297	Synergism
CC_50_	SLC	0.54866	Synergism	0.79139	Synergism
CC_50_	CC_50_	0.11558	Synergism	0.96751	Synergism
**ICRP**	**EPI**	**CEM**	**Interpretation**	**L5178Y-R**	**Interpretation**
SLC	CC_50_	0.06456	Synergism	0.08965	Synergism
CC_20_	CC_20_	0.08239	Synergism	0.86136	Synergism
CC_20_	CC_50_	0.02441	Synergism	0.00745	Synergism
CC_50_	SLC	0.47013	Synergism	0.76424	Synergism
CC_50_	CC_50_	0.06537	Synergism	0.01220	Synergism
**ICRP**	**ETO**	**CEM**	**Interpretation**	**L5178Y-R**	**Interpretation**
SLC	CC_50_	0.69184	Synergism	0.06804	Synergism
CC_20_	CC_20_	0.47243	Synergism	0.02997	Synergism
CC_20_	CC_50_	0.03856	Synergism	0.03988	Synergism
CC_50_	SLC	0.23747	Synergism	0.30940	Synergism
CC_50_	CC_50_	0.26008	Synergism	0.01571	Synergism

CI < 1 represents synergism; CI = 1 is additive effect; and CI > 1 indicates antagonism.

**Table 2:** DRI values compilation from the combinations of ICRP with chemotherapies in tumoral T-cell lymphoblasts.

Cytotoxic Concentration		Drug Reduction Index (DRI) for the Chemotherapies			
**ICRP**	**CTX**	**CEM**	**Interpretation**	**L5178Y-R**	**Interpretation**
SLC	CC_50_	8.42954	Favorable	31.1687	Favorable
CC_20_	CC_20_	17.5163	Favorable	18.5702	Favorable
CC_20_	CC_50_	54.1691	Favorable	67.9464	Favorable
CC_50_	SLC	34.3030	Favorable	39.9607	Favorable
CC_50_	CC_50_	80.3531	Favorable	24.2353	Favorable
**ICRP**	**DOX**	**CEM**	**Interpretation**	**L5178Y-R**	**Interpretation**
SLC	CC_50_	12.9454	Favorable	2.58459	Favorable
CC_20_	CC_20_	2.29273	Favorable	1.92477	Favorable
CC_20_	CC_50_	27.4377	Favorable	1.94132	Favorable
CC_50_	SLC	24.6198	Favorable	25.6457	Favorable
CC_50_	CC_50_	15.6821	Favorable	2.48874	Favorable
**ICRP**	**EPI**	**CEM**	**Interpretation**	**L5178Y-R**	**Interpretation**
SLC	CC_50_	20.4730	Favorable	14.0321	Favorable
CC_20_	CC_20_	71.7907	Favorable	2.87026	Favorable
CC_20_	CC_50_	340.605	Favorable	369.487	Favorable
CC_50_	SLC	194.782	Favorable	14.4692	Favorable
CC_50_	CC_50_	133.968	Favorable	369.487	Favorable
**ICRP**	**ETO**	**CEM**	**Interpretation**	**L5178Y-R**	**Interpretation**
SLC	CC_50_	1.59690	Favorable	21.5251	Favorable
CC_20_	CC_20_	13.5184	Favorable	1139.71	Favorable
CC_20_	CC_50_	280.660	Favorable	171.687	Favorable
CC_50_	SLC	1582.31	Favorable	507.816	Favorable
CC_50_	CC_50_	19.9247	Favorable	1724.07	Favorable

DRI < 1 represents not favorable dose reduction; DRI = 1 is not dose reduction; and DRI > 1 indicates favorable dose reduction.

## Data Availability

Data is contained within the article or Supplementary Material.

## Supplementary Materials

The following supporting information can be downloaded at https://www.mdpi.com/article/10.3390/ijms25147938/s1.
