# Supplementary material for: Synergistic Enhancement of Chemotherapy-Induced Cell Death and Antitumor Efficacy against Tumoral T-Cell Lymphoblasts by IMMUNEPOTENT CRP
Source: Int J Mol Sci. 2024 Jul 20;25(14):7938. doi: 10.3390/ijms25147938 (PMC11276711; doi:10.3390/ijms25147938)
Supplement: Supplementary file 1 [file ijms-25-07938-s001.zip › ijms-3030752-supplementary.pdf]

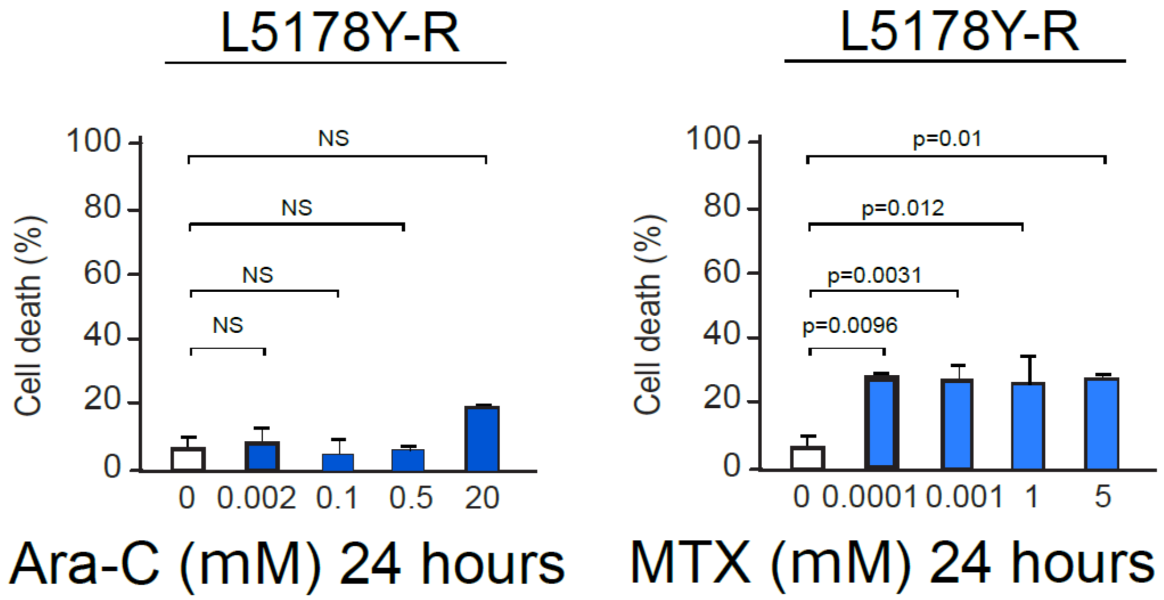

Figure S1: Cytarabine, Ara-C, (0.002, 0.1, 0.5, 20 mM) or Methotrexate, MTX, (0.0001, 0.001, 1, 5 mM)-treatment of L5178Y-R cells during 24 hours induce a non-significant (Ara-C) or low [<30%](MTX) cell death induction.
